# Vitiligo Following COVID-19 Vaccination and Primary Infection: A Case Report and Systematic Review

**DOI:** 10.7759/cureus.45546

**Published:** 2023-09-19

**Authors:** Lauren C Kasmikha, Meghan Mansour, Samantha Goodenow, Steven Kessler, Joel Appel

**Affiliations:** 1 Internal Medicine, Wayne State University School of Medicine, Detroit, USA; 2 Dermatology, Oakland University William Beaumont School of Medicine, Rochester Hills, USA; 3 Family Medicine, Wayne State University School of Medicine, Detroit, USA; 4 Internal Medicine, Oakland University William Beaumont School of Medicine, Rochester Hills, USA; 5 Internal Medicine/ Hematology-Oncology, Detroit Medical Center Sinai Grace Hospital, Detroit, USA

**Keywords:** post-covid 19 vaccine complications, covid-19 vaccine complication, covid-19 related immune dysregulation, covid-19 vaccine, infection, autoimmune disease and covid-19, vitiligo, covid-19

## Abstract

Vitiligo is an acquired pigmentation disorder with different theorized etiologies, although the exact pathogenesis is still largely unknown. It presents as well-demarcated white plaques throughout the body that result from the loss of melanocytes within the epidermis. Commonly, this condition presents alongside other autoimmune conditions, and it is associated with both genetic and non-genetic factors. We present a patient with no history of autoimmune disease who developed vitiligo after receiving her vaccines against COVID-19. This first occurred within 24 hours of receiving her first vaccine and then worsened after receiving her second vaccine. The depigmented rash was localized to the face, arms, and chest. She was treated with both oral and topical steroids, as well as topical tacrolimus cream. Despite adherence to treatment, the patient only reported subjective improvement in her skin lesions overall. While vitiligo arises sporadically, the temporal relationship between vaccinations and depigmentation makes a stronger case for the vaccine as the inciting factor for this patient, though coincidence is possible. A systematic review of the literature regarding the onset of vitiligo following both infection with and vaccination against COVID-19, this case offers a unique presentation that had a sudden onset and creates a learning opportunity for clinicians to investigate the potential relationship between the receipt of the vaccine and the onset of this skin condition. The goal of this report is to help clinicians be cognizant of the possibility of developing or worsening skin diseases after infection or vaccination so that they can be addressed and treated appropriately.

## Introduction

Vitiligo affects 0.5%-2% of the world population, classically presenting as well-demarcated hypopigmented patches on the body [1]. The pathogenesis is thought to be genetic and autoimmune-driven, resulting in the destruction of melanocytes [1]. Viral infections have been proposed among several identified triggers to play a significant role in vitiligo’s etiology; however, vaccines against COVID-19 have not been reported to date as a definitive causative factor. However, there is a paucity of literature describing the role of COVID-19 infection or vaccination. To our knowledge, there have been nine other reported cases of this unique cutaneous complication. Here we report another sudden-onset case of vitiligo after vaccination against COVID-19. In addition, we have systematically reviewed the literature regarding the onset of vitiligo following infection with and vaccination against COVID-19.

## Case presentation

**Figure 1:**
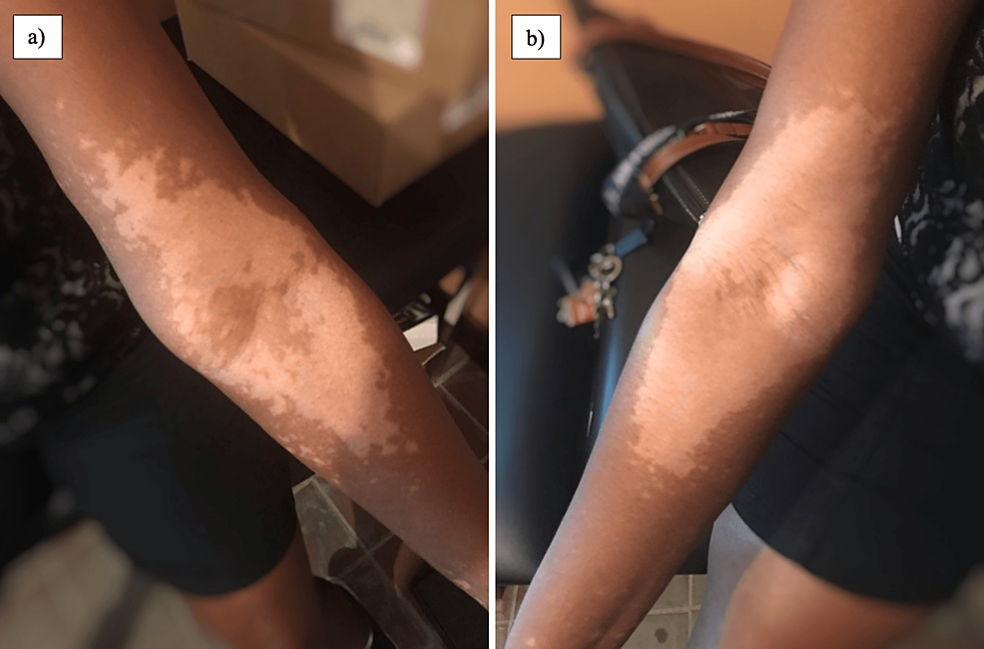
Appearance of the left antecubital fossa (Figure 1A) and right antecubital fossa (Figure 1B) following the second vaccination dose, early in the disease course

**Figure 2:**
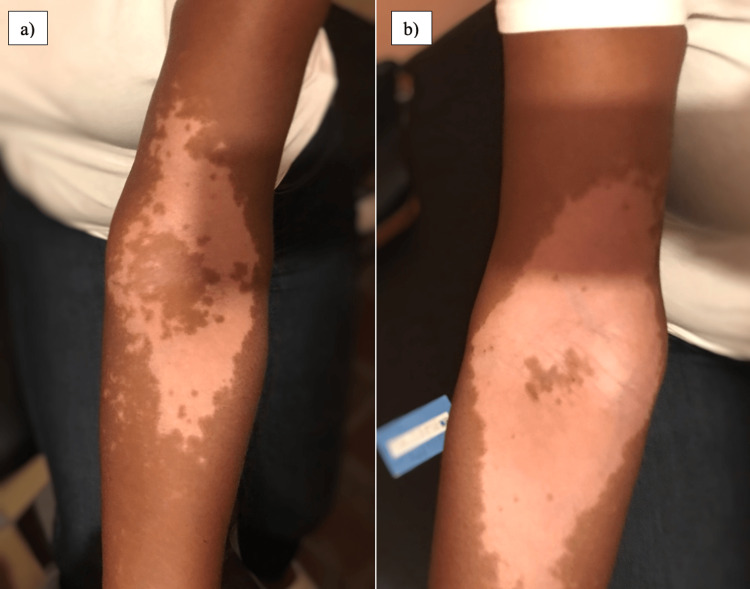
Appearance of the left antecubital fossa (Figure 2A) and right antecubital fossa (Figure 2B) weeks after beginning immunosuppressive therapy, with some noted improvement

A 62-year-old female with a medical history of hypertension managed with amlodipine presented with a chief complaint of a rash on her face, neck, chest, and arms. This occurred within 24 hours of having received the Pfizer-BioNTech SARS-CoV-2 vaccine. She denied pain or pruritus. Subsequently, after receiving her second dose of the vaccine, she noted an increase in the extent of the rash. Further history was negative for a personal or familial history of autoimmune disorders. A physical exam revealed a depigmented rash on her head and neck, and further examination of the skin demonstrated similar findings on her chest and arms. There was no evidence of erythema, purulence, maculopapular changes, or excoriation. The remainder of the exam was unremarkable. Management included topical triamcinolone cream 2% and tacrolimus cream. Additional therapy included oral dexamethasone. Figures 1A, 1B depict the appearance after the second dose, and Figures 2A, 2B show the appearance of the rash after topical and oral therapy were administered.

She reported adherence to her treatment and noted some subjective improvement but no significant resolution overall.

## Discussion

Vitiligo is an acquired pigmentation disorder characterized histologically by the loss of epidermal melanocytes [2]. It typically presents as white, depigmented skin lesions. Though the exact etiology is unknown, vitiligo has been commonly attributed to provoking events like physical injury or emotional stress. Further, patients with comorbid autoimmune diseases have been noted to more frequently develop vitiligo.

**Figure 3:**
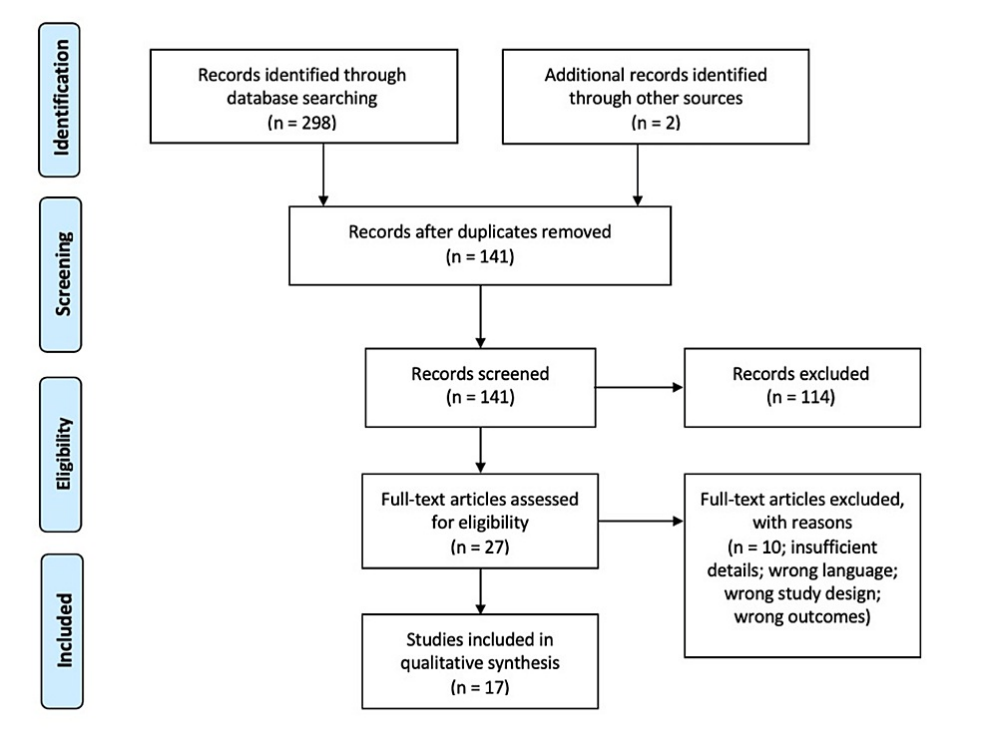
Article selection reported via the Preferred Reporting Items for Systematic Reviews and Meta-Analyses (PRISMA) diagram.

While vitiligo can arise sporadically in many individuals, the temporal relationship between the two Pfizer vaccinations and the development of depigmentation makes a stronger case for the vaccine as the inciting factor for this patient. We reviewed the literature regarding both the COVID-19 primary infection and vaccine prophylaxis as triggers for the onset of vitiligo skin lesions. Four unique databases- PubMed, Embase, Scopus, and Web of Science-were searched for articles pertaining to the onset or worsening of vitiligo following COVID-19 infection or vaccination. Of the 141 articles screened, 15 met the inclusion criteria and were included in the study (Figure 3).

The authors established the quality of evidence using the 2009 Oxford Levels of Evidence criteria, presented in Table 1.

**Table 1:** Quality of evidence as established by the 2009 Oxford Levels of Evidence Criteria

Author	Level of Evidence
Herzum et al. [3]	4
Gamonal et al. [4]	4
Schmidt et al. [5]	4
Kaminetsky J, Rudikoff D [6]	4
Bukhari AE [7]	4
Flores-Terry et al. [8]	4
Aktas H, Ertuğrul G [9]	4
Koç Yıldırım S [10]	4
Singh et al. [11]	4
Nicolaidou et al. [12]	4
López et al. [13]	4
Uğurer et al. [14]	4
Ciccarese et al. [15]	4
Militello et al. [16]	4
Macca et al. [17]	4
Caroppo et al. [18]	4
Okan G, Vural P [19]	4

A total of 17 patients were identified; 15 cases (88.2%) with new-onset or worsening vitiligo after COVID-19 vaccination and two cases (11.8%) of vitiligo following primary COVID-19 infection. A majority of the patients (15, 88.2%) reported a primary episode of vitiligo following the COVID-19 vaccination or infection. The remaining patients (two, 11.8%) reported worsening of previously diagnosed vitiligo that may be considered Koebnerization. Clinicians diagnosed vitiligo via Wood’s lamp (13, 76.5%), biopsy (two, 11.8%), Wood’s lamp and biopsy (one, 5.9%), or solely clinically (one, 5.9%). Depigmentation occurred in various locations, and 11 (64.7%) patients reported generalized vitiligo, while six (35.3%) patients reported local vitiligo. In regard to the cases following vaccination, eight cases (53.3%) described hypopigmented lesions following the first dose of the vaccine, and six cases (40.0%) described the onset following the second dose. Of the total, only four patients (23.5%) reported a history of autoimmune disease. Several therapies were used to treat the lesions, with resolution documented as follows: complete improvement (0, 0.0%), some improvement (four, 23.5%), no improvement (two, 11.8%), and no follow-up reported (11, 64.7%). Pertinent clinical characteristics, patient demographics, and outcomes are illustrated in Table 2.

**Table 2:** Patient demographics, clinical characteristics, and outcomes of COVID-19-associated vitiligo * n adds to more than 17 cases because some patients fell within more than one category. ** n adds to 15 cases because two cases were primary infections and thus, not included.

	(n)	%
Sex		
Male	7	41.2
Female	10	58.8
Average age (years)	50	
Average time to presentation (days)	11	
First episode vs. Koebnerization		
First episode	15	88.2
Koebnerization	2	11.8
Vitiligo diagnosis		
Wood’s lamp	13	76.5
Biopsy	2	11.8
Wood’s lamp and biopsy	1	5.9
Clinical only	1	5.9
Generalized vs. local vitiligo		
Multiple bodily locations	11	64.7
One bodily location	6	35.3
Location of vitiligo*		
Face	11	64.7
Trunk	9	52.9
Upper extremities	6	35.3
Neck	4	23.5
Hands	4	23.5
Lower extremities	3	17.6
Location of trauma (injection or biopsy)	2	11.8
Genital area	2	11.8
Scalp	1	5.9
Primary infection vs. vaccine		
Primary infection	2	11.8
Vaccine	15	88.2
Vaccine manufacturer**		
Pfizer	8	53.3
Moderna	3	20.0
Other	4	26.7
Dose of vaccine**		
First	8	53.3
Second	6	40.0
Not reported	1	6.7
Personal history of autoimmune disease		
Yes	4	23.5
No	11	64.7
Not reported	2	11.8
Family history of autoimmune disease		
Yes	2	11.8
No	9	52.9
Not reported	6	35.3
Family history of vitiligo		
Yes	2	11.8
No	11	64.7
Not reported	4	23.5
Therapy combinations		
Three agents	3	17.6
Two agents	2	11.8
One agent	11	64.7
Not reported	1	5.9
Treatment*		
Topical calcineurin inhibitor	10	58.8
Topical steroid	6	35.3
Phototherapy	4	23.5
Oral steroid	2	11.8
Topical khellin (herb)	1	5.9
Not reported	1	5.9
Outcome		
Complete improvement	0	0.0
Some improvement	4	23.5
No improvement	2	11.8
Not reported	11	64.7

The prevailing autoimmune theory postulates that the destruction of melanocytes is secondary to the activation of cytotoxic T-cells [1-2]. Biopsies of perilesional areas of vitiligo have demonstrated infiltrating cytotoxic CD8+ lymphocytes that can cause apoptosis of melanocytes [1]. Several cytokines have also been implicated in melanocyte destruction, including elevated levels of tumor necrosis factor-alpha (TNF-α), interferon-gamma, and interleukin-10 and-17 [1]. Interestingly, the Pfizer-BioNTech vaccine has also been linked to a similar inflammatory response. This response manifests as an upregulation of T-helper 1 (Th1) cells, leading to increased interleukin-2, interferon-gamma, and TNF-α, which may be involved in the pathogenesis of vitiligo [1,3].

There have been several reported cases of new-onset autoimmune diseases following vaccination against COVID-19, including lichen planus, Guillain-Barré syndrome, systemic lupus erythematosus, and immune thrombotic thrombocytopenia [3, 20]. Notably, one case included in our study described an eruption of lichen planus after the first dose of the COVID-19 vaccine, which worsened after the second dose. Additionally, the patient developed vitiligo macules in several areas after the second dose [4]. Severe infection with COVID-19 can lead to immune dysregulation of CD8+ T lymphocytes and subsequent immune reconstitution. The drastically disordered immune responses can then lead to increased oxidative stress and the eventual development of a "cytokine storm," in which there are excess inflammatory cytokines and overactivation of immune cells [5]. It is possible that a similar pathogenesis can follow vaccination and lead to similar inflammatory responses. Another suggested theory for the autoimmune etiology of vitiligo following COVID-19 infection and vaccination includes the phenomenon of molecular mimicry, in which autoantibodies or vaccine adjuvants can contribute to autoimmunity [20].

## Conclusions

While vitiligo can arise sporadically, the temporal relationship between the two Pfizer vaccinations and the development of depigmentation makes a stronger case for the vaccine as the inciting factor for this patient. It is possible that the inflammatory response to this vaccine could potentially trigger emerging autoimmune reactions. The small sample size and heterogeneity of reported data limit our study. While there are several reports of vitiligo following COVID-19 infection or vaccination, including our own, it is plausible that the viral trigger is merely a coincidence, especially considering the few reported cases. While extremely rare, it is important to be cognizant of the possibility of developing or worsening skin diseases after the COVID-19 vaccination or infection. However, we believe this risk of skin disease is far outweighed by the benefit of receiving vaccination-conferred protection against hospitalization and the risk of death resulting from severe COVID-19 infections. Further investigation is needed to explore the potential link and establish a causal relationship between the COVID-19 vaccination and the onset of vitiligo and other autoimmune conditions. Meanwhile, clinicians should be aware of the possibility of similar reactions and treat them appropriately.
